# Utilization of Food-Derived β-Glucans to Prevent and Treat Non-Alcoholic Fatty Liver Disease (NAFLD)

**DOI:** 10.3390/foods12173279

**Published:** 2023-09-01

**Authors:** Nelson Kei, Vincent Wai Sun Wong, Susana Lauw, Lijun You, Peter Chi Keung Cheung

**Affiliations:** 1 Food and Nutritional Sciences Program, School of Life Sciences, The Chinese University of Hong Kong, Hong Kong SAR, China; nelsonkei@link.cuhk.edu.hk (N.K.); susana.lauw@link.cuhk.edu.hk (S.L.); 2 Department of Medicine and Therapeutics, The Chinese University of Hong Kong, Hong Kong SAR, China; wongv@cuhk.edu.hk; 3 School of Food Science and Engineering, South China University of Technology, Guangzhou 510640, China; feyoulijun@scut.edu.cn

**Keywords:** β-glucan, natural polysaccharides, non-alcoholic fatty liver disease (NAFLD), non-alcoholic steatohepatitis (NASH)

## Abstract

Non-alcoholic fatty liver disease (NAFLD) has become the most common chronic liver disease nowadays. Currently, there is no officially approved drug to treat NAFLD. In view of the increasing global prevalence of NAFLD and an absence of treatments, the development of effective treatments is of utmost importance. β-glucan, a natural bioactive polysaccharide, has demonstrated hepatoprotective effects in NAFLD prevention and treatment. This review solely focuses on gathering the published preclinical animal studies that demonstrated the anti-liver injury, anti-steatotic, anti-inflammatory, anti-fibrotic, and antioxidant activities of β-glucan. The impact of β-glucan on gut microbiota and its metabolites including short-chain fatty acids and bile acids as the underlying mechanism for its bioactive beneficial effect on NAFLD is also explored. Given the limited knowledge of β-glucan on anti-fibrotic activity, bile acid metabolism, and gut microbiota function, additional relevant research is highly encouraged to lay a solid foundation for the use of food-derived β-glucan as a functional food for NAFLD. It is envisaged that further investigation of food-derived β-glucan in human clinical studies should be carried out for its wider utilization.

## 1. Introduction

Non-alcoholic fatty liver disease (NAFLD) presently stands as the prevailing chronic liver disease in the world, which is often attributed to an unhealthy diet and a sedentary lifestyle because of urbanization [1]. Non-alcoholic steatohepatitis (NASH), the more severe form of NAFLD, has been identified as a growing indication for liver transplantation in the United States [2], Italy [3], and Nordic countries [4]. The identification of NAFLD as the hepatic manifestation of metabolic syndrome (MetS) is widely investigated, with its characteristic features encompassing abdominal obesity, high blood pressure, dyslipidemia, and hyperglycemia [5]. Recently, a systematic review and meta-analysis shows that the worldwide prevalence of NAFLD is estimated to be 38% [6]. However, NAFLD is more prevalent among individuals with MetS traits and type 2 diabetes [7], which account for greater than 70% and approximately 56%, respectively [8,9]. NAFLD comprises non-alcoholic fatty liver (NAFL) and NASH. NAFL, also known as hepatic steatosis, is characterized by the presence of intrahepatic triglycerides that account for at least 5% of liver weight or lipid vacuoles found in 5% of hepatocytes by imaging or histology [10,11]. Although NAFL is relatively benign [12], 20% of the population with NAFL could progress to NASH [13]. NASH is different from NAFL due to the presence of hepatic inflammation and hepatocyte ballooning on top of hepatic steatosis [7]. NASH could progress to fibrosis manifesting at different stages, cirrhosis, and hepatocellular carcinoma [14]. Liver fibrosis has been reported to be the key prognostic marker for the risk of death in NAFLD patients [15,16]. It is essential to exclude factors such as excessive alcohol intake, parenteral nutrition, genetic influences, or the use of steatogenic drugs to diagnose NAFLD [17].

Although dietary and behavioral modification is the primary recommended therapy for NAFLD, they are difficult to practice due to the lack of guidance [18]. In the absence of approved medication, a highly practical yet effective solution to treat NAFLD is in urgent demand. Recent evidence has increasingly highlighted the contributory role of the gut microbiome in the development of NAFLD [19]. The development of NAFLD has been accompanied by the diminishment of microbiome diversity in humans and mice [20,21]. Oral gavage of high-alcohol-producing *Klebsiella pneumoniae* isolated from a NAFLD individual induced the onset of fatty liver in mice because of the elevated hepatic uptake of endogenous alcohol produced by this bacterium [22]. Study on the role of gut-liver axis in NAFLD development has increasingly gained attention [23]. Along this axis, the gut and its microbiota as well as the liver interact with each other in a bidirectional manner through the portal vein [24]. Damaged gut barrier function due to dysbiosis could facilitate the translocation of harmful bacteria and metabolites from the gut to the liver, intensifying NAFLD [25]. Prevention and management of NAFLD might be achieved by targeting the gut-liver axis using natural polysaccharides via protecting the gut barrier and reprogramming the gut microbiota and its metabolites [25].

Recently, β-glucan was reviewed as a dietary fiber that is beneficial to lipid and glucose metabolism while improving the gut microbiota composition during the state of metabolic diseases [26,27]. Provided the fact that accumulation of hepatic triglycerides and inflammation are the key phenomenon in NAFLD, the application of β-glucan as a functional food to prevent and improve NAFLD is practical because of its hypotriglyceridemic and anti-inflammatory properties [28]. β-glucans are a group of non-digestible polysaccharides containing D-glucose monomers connected with either β-1,3-1,4 or β-1,3-1,6 glycosidic linkages that can be naturally found in the cell wall of yeast, bacteria, mushrooms, seaweed, and cereals [29]. Apart from its health-promoting potential, β-glucans could act as a novel source of prebiotics [30]. Prebiotic is defined as “a substrate that is selectively utilized by host microorganisms conferring a health benefit” [31]. On the other hand, probiotic is defined as “live microorganisms which when administered in adequate amounts confer a health benefit on the host” [32]. There are accumulating in vitro and in vivo studies showing an enrichment of probiotic *Bifidobacteria* driven by β-glucans [28]. In an in vitro study, β-glucans could serve as primary carbon source for fermentation by *Bifidobacterium longum* subsp. *Infantis* [33]. Barley β-glucan has been reported to have a significant bifidogenic effect in healthy individuals aged between 50 and 70 years old [34]. It is likely that β-glucans are beneficial to the gut microbiota by increasing the abundance of probiotic bacteria. Over the past decade, β-glucans have been growingly studied for their anti-NAFLD effects in animal models. This review specifically gathered current scientific evidence on the utilization of food-derived β-glucans in preclinical animal models.

## 2. Effects of Food-Derived β-Glucans on NAFLD

To our knowledge, published human data on the effects of food-derived β-glucans on NAFLD patients are rare. Therefore, clinical trials are not considered in this review. The key study findings are summarized in Table 1, providing information on the type of β-glucan, characteristics of the study approach, dosage and study period of the β-glucan interventions, purity of β-glucan employed along with the primary effects observed from the interventions. Table 1 aims to provide readers with an overview of the effects of β-glucan on NAFLD in preclinical animal studies and it could help researchers to design additional experiments to elucidate the bioactivity mechanisms of different food-derived β-glucans that are yet to be known.

The majority of research works included in Table 1 show that β-glucan possesses anti-liver injury, anti-steatotic, anti-inflammatory, anti-fibrotic, and antioxidant activities. β-glucan derived from cereals and yeasts are frequently studied in the context of NAFLD. Only a few studies evaluated the impact of β-glucan on gut microbiota and its metabolites. Investigations in animal models are commonly conducted using a single source of β-glucan and from a prevention perspective instead of a treatment approach.

The purity of β-glucan is often not stated, which could affect the actual dosage of β-glucan supplementation. Most of the studies investigated the effect of β-glucan on the anti-NAFLD activities without a simultaneous assessment of the gut microbiota and its metabolites. Despite β-glucan could bring positive effects on the liver in 3 to 24 weeks, whether the improvement can be sustained after the termination of β-glucan supplementation has not been clarified.

### 2.1. Anti-Liver Injury Activity

Liver enzymes in the blood circulation including alanine aminotransferase (ALT) and aspartate aminotransferase (AST) are commonly used liver function markers to assess the extent of liver injury. Variation in the results of β-glucan originated from fungi was observed across different studies. Interestingly, a rise in serum ALT was demonstrated when male C57BL/6J male mice fed a high-fat diet (HFD) with 5 days of colitis induction using dextran sulfate sodium were supplemented with β-glucan produced from *Schizophyllum commune* for 12 weeks since baseline [42]. This suggests that there might be safety concerns to use β-glucan when there is concurrent onset of colitis and NAFLD. β-glucan derived from *Aureobasidium pullulans* has been shown to reduce serum liver enzymes in male C57BL/6N mice and male hamsters fed HFD starting from the beginning of the study in 16 weeks and 8 weeks, respectively [51,52]. Similarly, 8-week supplementation of baker’s yeast β-glucan could decrease the serum liver enzymes in male C57BL/6J male mice fed a methionine and choline-deficient diet (MCD) [45]. It was revealed that β-glucan obtained from *Antrodia cinnamomea* and *Lentinus edodes* could effectively suppress the level of liver enzymes in NAFLD-induced male C57BL/6 mice in 45 days and 15 weeks, respectively [36,44]. Additionally, Gao et al. reported that β-glucan from *Polyporus umbellatus* (*Pers.*) Fries sclerotia could reduce the serum level of ALT and AST in C57BL/6J mice in 3 weeks [35]. However, Ikewai et al. showed no significant change in plasma ALT when 2-week HFD-fed STAM^TM^ mice were supplemented with *A. pullulans* strain AFO-202 and/or N-163 β-glucan for 3 weeks compared to the control mice [38]. Moreover, 15-day supplementation of β-glucan produced by *Botryosphaeria rhodina* to 6-week high-fat and high-sugar diet-fed male Wistar rats provided with sucrose-containing water could not show a significant change in serum liver enzymes [50].

Cereal β-glucans have shown a more consistent trend of serum liver enzyme reduction in the prevention rodent models. For example, a reduction in ALT was seen in HFD-fed male albino rats and intraperitoneally injected lipopolysaccharide (LPS) male C57BL/6 mice supplemented with oat β-glucan for 24 weeks and 6 weeks, respectively [37,54]. Similar findings could be demonstrated in supplementation of highland barley β-glucan, which was found to decrease the levels of serum ALT and AST in male C57BL/6 mice fed the western diet and sugar-containing water [40]. Unfortunately, investigation on the anti-liver injury activity of cereal β-glucans in NAFLD-induced animals remains limited to date. The magnitude of liver enzyme reduction driven by β-glucan is between 15% and 60% in animal studies showing an improvement in liver injury, which holds the potential to translate into clinical benefits.

### 2.2. Anti-Steatotic Activity

Histopathological assessment of biopsy specimens has been regarded as the golden standard to diagnose NAFL and NASH [55]. Our selected studies performed either hematoxylin and eosin (H&E) or oil red O staining of the liver specimen to determine the change in histopathology. It appears that majority of the studies demonstrated that supplementation of β-glucan derived from cereal or fungi could prevent NAFL [37,40,42,45,46,49,51,52,54]. Although there are 3 studies using oat β-glucan displaying positive impact on the prevention of hepatic steatosis [37,48,49], Yau et al. reported no significant improvement in NAFL when oat β-glucan-containing HFD was consumed by male C57BL/6N mice for 17 weeks [47]. Currently, evaluation of the effect of β-glucan on NAFLD-induced animals is mainly focused on those originated from fungi. It has been shown that β-glucan obtained from *A. cinnamomea* [44], *A. pullulans* [38], *B. rhodina* [50], *L. edodes* [36], and *P. umbellatus* sclerotia could attenuate NAFL based on H&E-stained liver sections [35]. Although there is limited data available on the effectiveness of cereal β-glucan to reverse NAFL, Cheng et al. examined 10-week oat β-glucan supplementation in 14-week HFD-fed male C57BL/6J mice under circadian disruption [43]. They found that oat β-glucan could reverse the increase in hepatic lipid accumulation to a level comparable to that of the non-circadian disrupted mice [43].

To elucidate the possible bioactivity mechanism behind the improvement of hepatic steatosis observed in the specimens, hepatic gene and/or protein expression relevant to lipid metabolism would be performed. The development of NAFL could be attributed to abnormal hepatic usage or breakdown of lipids [56]. Du et al. reported that 15-week supplementation of β-glucan derived from *L. edodes* following 6-week NAFLD-induction by HFD not only could upregulate the hepatic expression of peroxisome proliferator-activated receptor α (PPARα) and carnitine palmitoyltransferase 1α (CPT1α) involved in fatty acid oxidation, but also inhibit the expression of cluster of differentiation 36 in the liver responsible for free fatty acid uptake [36,57]. Besides, hull-less barley β-glucan was found to suppress the hepatic expression of peroxisome proliferator-activated receptor γ (PPARγ), stearoyl-CoA desaturase 1, and lipoprotein lipase, resulting in a reduction in lipid synthesis and fatty acid uptake [46].

It is worth highlighting the crucial role of adenosine monophosphate-activated protein kinase (AMPK) activation in the alleviation of NAFL because it regulates the downstream genes involved in de novo lipogenesis [58]. In a study conducted by Liu et al., the prevention of NAFL due to oat β-glucan supplementation was contributed by the activation of the AMPK signaling pathway, which was accompanied by downregulation of hepatic lipogenic protein expression including phosphorylated acetyl-CoA carboxylase, fatty acid synthase and sterol regulatory element-binding protein 1 (SREBP1) [49]. An increased expression of hepatic protein PPARα and CPT1 was also observed [49]. Similar findings at the gene expression level could be demonstrated in a recent study using highland barley β-glucan as a preventive agent for NAFL [40]. Furthermore, β-glucan obtained from *A. cinnamomea* could upregulate sirtuin 1 and downregulate PPARγ and SREBP1c protein expression in the liver of NAFLD mice [44]. Taken together, the current evidence supports that β-glucan can be developed as a nutritional supplement to prevent or alleviate NAFLD.

### 2.3. Anti-Inflammatory Activity

Among the selected articles, there are only a few studies that were able to illustrate the improvement of NASH resulted from β-glucan supplementation visualized and quantified by H&E staining and the level of pro-inflammatory mediators, respectively. Recently, β-glucan from *P. umbellatus* sclerotia was reported to ameliorate hepatic inflammation induced by MCD via downregulation of pro-inflammatory and macrophage-associated factors in 3 weeks [35]. Additionally, β-glucan obtained from *A. cinnamomea* was found to alleviate NASH in male C57BL/6 mice fed a high-fat and high-fructose diet after 45 days [44]. Although the gene expression of pro-inflammatory cytokine interleukin-6 (IL-6) in the liver was not affected by supplementation of *A. pullulans* strain AFO-202 or N-163, the combination of these 2 strains led to downregulation of IL-6 in STAM™ mice [38]. It was shown that baker’s yeast β-glucan reduced hepatic inflammation and the expression of tumor necrosis factor-α (TNF-α) and IL-6 in MCD-fed mice using immunohistochemistry assay [45]. Further analysis revealed that suppression of endoplasmic reticulum (ER) stress by lowering glucose-regulated protein 78, phosphorylated eukaryotic translation initiation factor 2α, and phosphorylated c-Jun N-terminal kinase while increasing ER-resident protein 57, phosphorylated Ak strain transforming and phosphorylated mitogen-activated protein kinase was a possible bioactivity mechanism of baker’s yeast β-glucan in NASH prevention [45].

Previously, oat β-glucan was tested for its potential for NASH prevention in LPS-intraperitoneally injected mice [54]. It was found that oat β-glucan suppressed the levels of hepatic TNF-α, IL-6, and interleukin-1β (IL-1β). Such anti-inflammatory effect possessed by oat β-glucan might be owing to the improved gut barrier function indicated by elevated serum glucagon-like peptide 2 and reduced inflammation-triggering endotoxemia [54]. Similar anti-inflammatory activity of oat β-glucan was revealed in several NAFLD prevention studies with a study period lasting from 8 to 24 weeks [37,48,49].

Ke et al. showed that 8-week supplementation of oat β-glucan could lower serum LPS-binding protein and downregulate hepatic TNF-α gene expression in C57BL/6J mice fed HFD [48]. Furthermore, a decrease in hepatic TNF-α and IL-6 was found after 6-week HFD-fed mice treated with β-glucan obtained from *L. edodes* for 15 weeks [36]. Given the fact that these 2 studies reported a reduction in gene expression of pro-inflammatory cytokines after β-glucan supplementation, infiltration of inflammatory cells in the H&E-stained liver tissue has not been observed in the control group [36,48]. Potential bias might be introduced due to the use of different diets to induce NAFLD in animal studies. In fact, the diet selected by the researchers could influence the severity of NASH in the animals. It has been suggested that HFD would drive hepatic steatosis but not induce inflammation or ballooning even under prolonged feeding in mice [59]. This might explain why some of our included studies adopting HFD could not display the inflammation feature in NASH in both the control and intervention groups. Hence, the evaluation of β-glucan on preventing or ameliorating liver inflammation using HFD sometimes could be difficult to achieve. In view of the limited number of investigations of β-glucan in a well-established NASH model, more animal studies with a specific focus on NASH prevention or treatment are still needed to assess the anti-inflammatory activity and its mechanism of β-glucan.

### 2.4. Anti-Fibrotic Activity

Currently, research on the anti-fibrotic activity of β-glucan in NAFLD animal models is limited to fungi-type. Supplementation of β-glucan obtained from *A. pullulans* strain N-163 and AFO-202 combined with N-163 could reduce hepatic fibrosis, which was illustrated by a decrease in the Sirius red-positive area [38]. On top of the reduced Sirius red-positive area, Gao et al. reported that β-glucan from *P. umbellatus* sclerotia suppressed the hepatic gene expression of α-smooth muscle actin and collagen type I alpha 1 [35]. It is recommended to investigate the anti-fibrotic activity of different β-glucans in the future to understand their potential to prevent cirrhosis and its complications.

### 2.5. Antioxidant Activity

The increased production of reactive oxygen species (ROS) resulted from an overload of free fatty acids to the liver could cause oxidative stress, which acts as an important accelerator of NAFLD progression from NAFL to NASH [60,61]. It has been revealed that oat β-glucan had antioxidant activity by significantly elevating the superoxide dismutase (SOD) activity in the liver of LPS-intraperitoneally injected mice [54]. Kanagasabapathy et al. reported that β-glucan-rich extract of *Pleurotus sajor-caju* could increase antioxidant defenses of the liver such as increasing the levels of glutathione peroxidase, catalase, and SOD while reducing lipid peroxidation [53]. In another study conducted by Du et al., β-glucan obtained from *L. edodes* was demonstrated to lessen oxidative stress mediated by decreased hepatic ROS production and strengthened antioxidant capacity including higher levels of SOD, malondialdehyde, 4-hydroxy-2-nonenal and glutathione/glutathione disulfide ratio along with downregulation of hepatic pro-oxidant enzyme NADPH oxidase 2 and 4 in the liver [36]. These anti-oxidative effects protected the NAFLD mice against hepatic apoptosis, as reflected by an increase in anti-apoptotic B-cell lymphoma 2 (Bcl2) and a reduction in pro-apoptotic cleaved-Caspase3 and Bcl-2-associated X protein level [36]. Collectively, β-glucan could be viewed as a potential antioxidant functional food to alleviate liver injury caused by oxidative stress in NAFLD.

### 2.6. Alteration of Gut Microbiota

In view of the accumulating evidence of the distinct gut microbiota profile of NAFLD in humans [62], more attention has been paid to the modulatory effects of β-glucans on the gut microbiota and its metabolites. β-glucan obtained from *S. commune* and *A. pullulans* strain N-163 in mice utilized for NAFLD prevention and treatment, respectively, were found to decrease the relative abundance of *Bilophila* [39,42], a bacterial genus identified to be elevated abundance in NAFLD patients [63]. Moreover, β-glucan as a potential prebiotic has been reported to increase the abundance of probiotic bacterial genus *Lactobacillus* via supplementation of *S. commune* and *A. pullulans* (AFO-202 + N-163 strain) β-glucan [39,42]. Although an increased abundance of *Lactobacillus* was observed in obese NAFLD patients [64], there is an increasing body of knowledge about *Lactobacillus* species functioning as a supplement to improve NAFLD in animals and humans [65]. Another study conducted by Tang et al. showed that the anti-steatotic activity of hull-less barley β-glucan in mice could restore the level of *Dubosiella* and *Faecalibaculum*, which were negatively correlated with serum triglycerides [46]. Furthermore, a higher *Turicibacter* and lower *Helicobacter* abundance were associated with decreased levels of pro-inflammatory factors including serum LPS, IL-1β, and TNF-α [46].

Erysipelotrichaceae is a bacterial family identified to decrease in abundance as the severity of NAFLD increases [66]. It has been reported that supplementation of oat β-glucan could increase the Erysipelotrichaceae abundance that was found to be depleted after HFD consumption in mice [48]. Additionally, targeted metabolites analysis revealed an elevated level of cecal short-chain fatty acids (SCFAs) including acetate, propionate, and butyrate [48]. Supplementation of SCFAs has been increasingly known for its potential to alleviate hepatic steatosis and inflammation in rodents [67]. An increase in cecal butyrate after oat β-glucan supplementation in mice was also reported by Cheng et al., which might be resulted from a rise in butyrate producers Ruminococcaceae and Lachnospiraceae [43]. However, no significant increase in cecal SCFAs was found after supplementation of β-glucan obtained from *S. commune* [42], suggesting that there could be differential effects among different β-glucans on SCFAs. Decreased species richness of the gut microbiota has been reported as a feature of NAFL and NASH patients [68]. Interestingly, the improvement of hepatic steatosis due to oat β-glucan supplementation was followed by an increase in species richness [43], indicating an attenuation of gut dysbiosis [69]. Taken together, it is likely that β-glucan supplementation could induce favorable modulation of gut microbiota associated with improvement of NAFLD.

### 2.7. Regulation of Bile Acid Metabolism

Bile acids (BAs) are signaling molecules that can interact with nuclear receptors to influence metabolic and inflammatory functions [70]. Higher levels of fecal total BAs, primary BAs and primary to secondary BAs ratio were observed in NASH patients compared to healthy individuals [71]. A low level of *Clostridium leptum* found in NASH patients could be a possible mechanism to explain the reduced conversion of primary to secondary BAs [71,72]. Only 3 studies of our selected articles evaluated the effect of β-glucans on BA metabolism. Oat β-glucan was found to prevent the increase in fecal total BAs and primary BA biosynthesis caused by HFD [48]. However, contradictory results were reported in other animal studies using different origins of β-glucans [41,51]. For example, it was shown that *A. pullulans*-derived β-glucan increased fecal BA production and the gene expression of hydroxymethylglutaryl-CoA reductase and cholesterol 7 alpha-hydroxylase (CYP7A1) in the liver [51]. Another study revealed that supplementation of highland barley β-glucan led to an increase in hepatic total BAs, primary BAs, and secondary BAs but a decrease in primary to secondary BAs ratio [41]. The increase in hepatic total BAs in the liver was attributed to elevated protein expression of CYP7A1, sterol 27-hydroxylase, and sterol 12 alpha-hydroxylase [41]. It was concluded that the highland barley β-glucan could prevent NAFL due to suppressed ileal farnesoid X receptor (FXR) activity, unregulated hepatic gene expression of enzymes involved in BAs synthesis, and activated hepatic FXR signaling [41]. Given the discrepancy between the anti-steatotic activity and the change in BA profile, a general idea of how β-glucan would affect the BA metabolism to prevent NAFLD based on current evidence could not be given. Therefore, additional evaluation of BA metabolism in animal studies is needed in the future.

## 3. Conclusions

**Figure 1:**
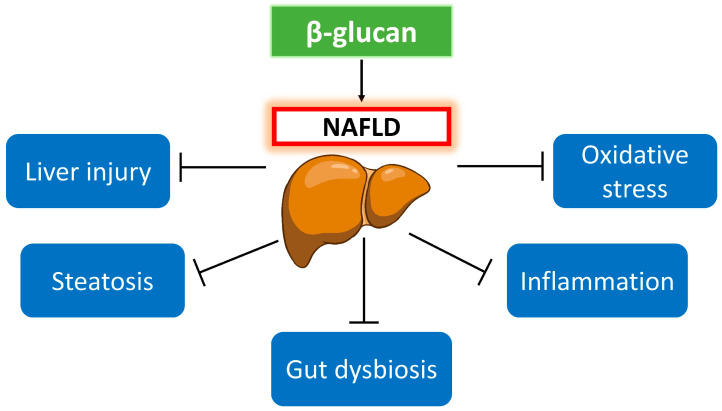
Beneficial effects of β-glucan on NAFLD.

β-glucan is a natural polysaccharide with a diverse range of bioactivities in the prevention and treatment of NAFLD. This review described the scientific evidence from animal studies supporting the beneficial effects of anti-liver injury, anti-steatosis, anti-inflammation, anti-fibrosis, and anti-oxidation. Moreover, β-glucan could modulate the gut microbiota and its metabolites such as SCFAs and BAs to improve NAFLD. The beneficial effects of β-glucan on NAFLD are illustrated in Figure 1. Since the studies included in this review are carried out on animals, translation of the efficacy of β-glucan from animals to humans may not be direct. Additionally, there is a great potential for β-glucan to be developed as a functional food for NAFLD. More mechanistic studies to explain the anti-NAFLD activities are needed. Further research on the anti-liver injury and anti-steatotic activities of β-glucan could be conducted among overweight and obese individuals who are likely to present NAFL. Although extensive studies have been conducted to illustrate the effectiveness of β-glucan to prevent NAFL, there are few research works on NASH prevention. The potential translation of the preclinical findings into clinical applications for NAFLD patients remains uncertain because of the limited number of both animal and human studies using β-glucan to treat NAFLD. Therefore, the effects of different types of β-glucan treatment in the early (NAFL) and later stage (NASH) of NAFLD could be compared before the launch of clinical trials for therapeutic success. Furthermore, a side-by-side comparative study on different β-glucan sources is highly recommended to offer clearer conclusions of their efficacy. In addition to β-glucan derived from cereals and fungi, those obtained from seaweed should be investigated in the context of NAFLD to provide comprehensive insights. We anticipate more clinical trials to be conducted in the future to establish direct benefits of β-glucan in NAFLD patients. Given the significance of gut microbiota in NAFLD progression, studying the effects of β-glucan on germ-free mice with NAFLD can clarify the role of gut microbiota in disease prevention and improvement. More in-depth research on the alteration of bacterial species and the function of gut microbiota due to β-glucan supplementation is required using shotgun metagenomic sequencing to clarify the underlying molecular mechanisms. This could foster the utilization of food-derived β-glucan as a carbohydrate-based prebiotic for NAFLD management in the future.

## Figures and Tables

**Table 1:** Effects of β-glucans on NAFLD in preclinical studies.

β-Glucan Type	Animal Model	Study Type	Intervention	Effects on Liver	Bioactivity Mechanisms	Reference
β-glucan from *Polyporus umbellatus* (*Pers.*) Fries sclerotia	C57BL/6J mice fed MCD	Treatment	Daily oral gavage Dose: 50 or 100 mg/kg BW Purity: NA Period: 3 weeks	↓ Serum ALT, AST ↓ Hepatic TG ↓ Hepatic steatosis, NAS, Oil red O-positive area, Sirius red-positive area ↓ F4/80, CD68, IL-1β, TNF-α, αSMA-positive area	↓ Hepatic CIDEC, SREBP1, PLIN2, PPARγ, CPT1A ↓ Hepatic IL-1β, IL-6, TNF-α, F4/80, CD68, CD11b, CCL5 ↓ Hepatic αSMA, COL1A1	[35]
Lentinan (β-glucan obtained from *Lentinus edodes*)	Male C57BL/6J mice fed HFD	Treatment	Daily oral gavage Dose: 6 mg/kg BW Purity: NA Period: 15 weeks	↓ Serum ALT, AST ↓ Hepatic TG, TC, Oil red O-positive area	↓ Hepatic TNF-α, IL-6, CD36 ↑ Hepatic PPARα, ACAT, and CPT1α ↓ Hepatic ROS, MDA, 4-HNE ↑ Hepatic GSH/GSSG, SOD1, SOD2 ↓ Hepatic NOX2, NOX4 ↑ Hepatic Bcl-2 ↓ Hepatic Caspase3 activity ↓ Hepatic cleaved-Caspase3, Bax	[36]
Oat β-glucan	Male albino rats fed HFD	Prevention	Mixed in diet Dose: 61.4 g/kg diet Purity: NA Period: 24 weeks	↓ Serum ALT, AST ↓ Hepatic steatosis, inflammatory infiltration	↓ Serum TMAO ↑ Hepatic GPR43	[37]
*Aureobasidium pullulans* strain AFO-202 β-glucan	STAM™ mice	Treatment	Daily oral administration Dose: 1 mg/kg BW Purity: NA Period: 3 weeks	NS Liver weight NS Plasma ALT NS Hepatic FFA ↓ NAS NS Sirius red, F4/80-positive area	NS Hepatic IL-6 ↓ Firmicutes, Enterobacteriaceae	[38,39]
*Aureobasidium pullulans* strain N-163 β-glucan	STAM™ mice	Treatment	Daily oral administration Dose: 1 mg/kg BW Purity: NA Period: 3 weeks	NS Liver weight NS Plasma ALT NS Hepatic FFA ↓ NAS ↓ Sirius red-positive area NS F4/80-positive area	NS Hepatic IL-6 ↓ *Bilophila*, *Turicibacter*	[38,39]
AFO-202 β-glucan + N-163 β-glucan	STAM™ mice	Treatment	Daily oral administration Dose: 1 mg/kg BW AFO-202 + 1 mg/kg BW N-163 β-glucan Purity: NA Period: 3 weeks	↓ Liver weight NS Plasma ALT NS Hepatic FFA ↓ NAS ↓ Sirius red-positive area NS F4/80-positive area	↓ Hepatic IL-6 ↓ Proteobacteria, *Prevotella* ↑ *Lactobacillus*	[38,39]
Highland barley β-glucan	Male C57BL/6 mice fed the western diet and 4.2% sugar water containing 18.9 g/L sucrose and 23.1 g/L fructose	Prevention	Daily oral administration Dose: 100 or 300 mg/kg BW Purity: 70% Period 16 weeks	100 mg/kg BW: ↓ Lipid droplets NS Liver index NS Serum ALT, AST ↓ Hepatic FFA NS NAS NS Hepatic TC, TG 300 mg/kg BW: ↓ Lipid droplets ↓ Liver index ↓ Serum ALT, AST ↓ NAS score ↓ Hepatic TC, TG, FFA	300 mg/kg BW: ↓ Hepatic FAS, SREBP1c ↑ Hepatic AMPKα, PPARα, CPT1α, CYP7A1, CYP27A1, CYP8B1, FXR, SHP ↑ Hepatic total BAs, primary BAs, secondary BAs ↓ Hepatic primary BAs/ secondary BAs ↑ Hepatic LCA, ApoCA, HDCA, DCA, UCA, TDCA, HCA ↑ Energy expenditure ↓ Ileal FXR ↑ Ileal FGF15	[40,41]
β-glucan produced from *Schizophyllum commune*	Male C57BL/6J male mice fed HFD + 5 days DSS-colitis induction	Prevention	Mixed in diet Dose: 3 g/kg HFD Purity: 95% Period: 12 weeks	↑ Serum ALT ↓ Hepatic steatosis grade, ratio of lipid droplet area	NS Cecal acetate, propionate, butyrate ↑ *Lactobacillus*, *Enterococcus* ↓ Lachnospiraceae, Ruminococcaceae, *Bacteroides, Bifidobacterium*, *Bilophila*, *Olsenella* ↓ Colon COX2	[42]
Oat β-glucan	Male C57BL/6J mice fed HFD	Treatment	Dissolved in drinking water Dose: 5% Purity: 75.3% Period: 10 weeks	↓ Hepatic steatosis	↑ Microbial species richness (ASV and Shannon diversity) ↑ Butyrate producers Lachnospiraceae, Ruminococcaceae ↑ Cecal butyrate and propionate	[43]
Antrodan (β-glucan obtained from *Antrodia cinnamomea*)	Male C57BL/6 mice fed a high-fat and high-fructose diet	Treatment	Daily oral gavage Dose: 20 or 40 mg/kg BW Purity: NA Period: 45 days	Both doses: ↓ Plasma ALT, AST, fat droplets, hepatic inflammation 40 mg/kg BW: ↓ Liver weight, liver weight to body weight ratio	↑ Hepatic Sirt1, p-AMPK ↓ Hepatic PPARγ, SREBP1c	[44]
Baker’s yeast β-glucan	Male C57BL/6J male mice fed MCD	Prevention	Daily oral administration Dose: 10, 30 or 100 mg/kg BW Purity: 90% Period: 8 weeks	10 mg/kg BW: NS Serum ALT, AST, oil red O-positive area, hepatic TC, ballooning score, liver weight, liver index ↓ Hepatic TG ↓ Steatosis, inflammation score, NAS 30 mg/kg BW: ↓ Serum ALT, AST ↓ Oil red O-positive area ↓ Hepatic TG, liver index NS Hepatic TC, ballooning score, liver weight ↓ Steatosis, inflammation score, NAS 100 mg/kg BW: ↓ Serum ALT, AST ↓ Oil red O-positive area ↓ Hepatic TG, liver index NS Hepatic TC, ballooning score, liver weight ↓ Steatosis, inflammation score, NAS	10 mg/kg BW: ↓ Hepatic TNF-α, IL-6 ↓ Hepatic p-JNK NS Hepatic GRP78, p-eiF-2α, p-MAPK ↑ Hepatic ERp57, p-Akt 30 mg/kg BW: ↓ Hepatic TNF-α, IL-6 ↓ Hepatic GRP78, p-eiF-2α, p-JNK ↑ Hepatic ERp57, p-Akt, p-MAPK 100 mg/kg BW: ↓ Hepatic TNF-α, IL-6 ↓ Hepatic GRP78, p-eiF-2α, p-JNK ↑ Hepatic ERp57, p-Akt, p-MAPK	[45]
Hull-less barley β-glucan	Male C57BL/6J mice fed HFD	Prevention	Daily oral gavage Dose: 500 mg/kg BW Purity: NA Period: 12 weeks	↓ Liver weight, lipid accumulation, hepatic TG	↓ Hepatic PPARγ, SCD1, LPL ↑ Hepatic HSL ↓ Serum LPS ↑ *Dubosiella*, *Faecalibaculum*, *Turicibacter* ↓ *Helicobacter*	[46]
Oat β-glucan	Male C57BL/6N mice fed HFD	Prevention	Mixed in diet Dose: 32 mg/day Purity: NA Period: 17 weeks	NS Hepatic steatosis, relative liver weight	NA	[47]
Oat β-glucan	Male C57BL/6J mice fed HFD	Prevention	Daily oral gavage Dose: 1 g/kg BW Purity: 80% Period: 12 weeks	↓ Hepatic steatosis	↓ Serum LBP ↓ Hepatic TNF-α ↑ Erysipelotrichaceae ↑ Cecal acetate, propionate, butyrate ↓ Fecal BA ↓ Fecal TCDCA, THDCA, TUDCA, CA, αTMCA, βTMCA, TCA	[48]
Oat β-glucan	Male ICR mice fed HFD	Prevention	Daily intragastric administration Dose: 500 or 1000 mg/kg BW Purity: 99% Period: 10 weeks	↓ Liver weight, lipid droplet, hepatic inflammation	500 mg/kg BW: NS Hepatic FAS ↑ Hepatic p-AMPKα, p-ACC, PPARα, CPT1 ↓ Hepatic SREBP1 1000 mg/kg BW: ↑ Hepatic p-AMPKα, p-ACC, PPARα, CPT1 ↓ Hepatic SREBP1, FAS	[49]
Botryosphaeran (β-glucan produced by *Botryosphaeria rhodina*)	Male Wistar rats fed a high-fat and high-sugar diet and water containing sucrose (300 g/L)	Treatment	Daily oral gavage Dose: 12 mg/kg BW Purity: NA Period: 15 days	NS Serum ALT, AST ↓ Liver weight, lipid quantity ↓ Hepatic TG	NA	[50]
*Aureobasidium pullulans*-derived β-glucan	Male C57BL/6N mice fed HFD	Prevention	Dissolved in drinking water Dose: NA Purity: 99% Period: 16 weeks	↓ Serum ALT ↓ Oil red O-positive area, hepatic TG, hepatic fat area revealed by CT scan	↑ Hepatic HMGR, CYP7A1 ↑ Fecal BA production	[51]
Polycan (β-glucan extracted from *A. pullulans*)	Male hamsters fed HFD	Prevention	Daily oral gavage Dose: 31.25, 62.5 or 125 mg/kg BW Purity: NA Period: 8 weeks	↓ Serum ALT, AST ↓ Hepatic fatty change region	NA	[52]
β-glucan-rich extract of *Pleurotus sajor-caju*	Female C57BL/6J mice fed HFD	Prevention	Oral gavage thrice a week Dose: 60, 120 or 240 mg/kg BW Purity: 80.55% Period: 16 weeks	60 mg/kg BW: NS Serum ALT ↓ Serum AST, ALP 120 or 240 mg/kg BW: ↓ Serum ALT, AST, ALP	↑ Hepatic GPx, CAT, SOD ↓ Hepatic LPO	[53]

↓: decrease; ↑: increase; 4-HNE: 4-hydroxy-2-nonenal; αSMA: α-smooth muscle actin; αTMCA: α-tauromuricholic acid; βTMCA: β-tauromuricholic acid; ACAT: acyl-coenzyme A:cholesterol acyltransferase; ALP: alkaline phosphate; ALT: alanine aminotransferase; AMPKα: adenosine monophosphate-activated protein kinase α; ApoCA: apocholic acid; AST: aspartate aminotransferase; ASV: amplicon sequence variant; BA: bile acid; Bax: Bcl-2-associated X; Bcl-2: B-cell lymphoma 2; BW: body weight; CA: cholic acid; CAT: catalase; CCL5: C-C motif chemokine ligand 5; CD11b: cluster of differentiation 11b; CD36: cluster of differentiation 36; CD68: cluster of differentiation 68; CIDEC: cell death-inducing DFFA-like effector c; COL1A1: collagen type I alpha 1; COX-2: cyclooxygenase-2; CPT1: carnitine palmitoyltransferase 1; CYP27A1: sterol 27-hydroxylase; CYP7A1: cholesterol 7 alpha-hydroxylase; CYP8B1: sterol 12 alpha-hydroxylase; DCA: deoxycholic acid; DSS: dextran sulfate sodium; ER: endoplasmic reticulum; ERp57: ER-resident protein 57; F4/80: epidermal growth factor module-containing mucin-like receptor 1; FAS: fatty acid synthase; FFA: free fatty acids; FGF15: fibroblast growth factor 15; FXR: farnesoid X receptor; GLP-2: glucagon-like peptide 2; GRP43: G-protein coupled receptor 43; GPx: glutathione peroxidase; GRP78: glucose-regulated protein 78; GSH: glutathione; GSSG: glutathione disulfide; HCA: hyocholic acid; HDCA: hyodeoxycholicacid; HFD: high-fat diet; HMGR: hydroxymethylglutaryl-CoA reductase; HSL: hormone-sensitive lipase; IL-1β: interleukin-1β; IL-6: interleukin-6; LBP: lipopolysaccharide-binding protein; LCA: lithocholic acid; LPL: lipoprotein lipase; LPO: lipid peroxidation; LPS: lipopolysaccharide; MCD: methionine and choline-deficient diet; MDA: malondialdehyde; NA: not available; NAS: NAFLD activity score; NOX: NADPH oxidase; NS: no significant effect; p-ACC: phosphorylated acetyl-CoA carboxylase; p-Akt: phosphorylated Ak strain transforming; p-AMPK: phosphorylated adenosine monophosphate-activated protein kinase; p-eiF-2α: phosphorylated eukaryotic translation initiation factor 2α; p-JNK: phosphorylated c-Jun N-terminal kinase; p-MAPK: phosphorylated mitogen-activated protein kinase; PLIN2: Perilipin 2; PPARα: peroxisome proliferator-activated receptor α; PPARγ: peroxisome proliferator-activated receptor γ; ROS: reactive oxygen species; SCD1: stearoyl-CoA desaturase 1; SHP: small heterodimer partner; Sirt1: sirtuin 1; SOD: superoxide dismutase; STAM: stelic animal model; SREBP1: sterol regulatory element-binding protein 1; TC: total cholesterol; TCA: taurocholic acid; TCDCA: taurochenodeoxycholic acid; TDCA: taurodeoxycholic acid; TG: triglycerides; THDCA: taurohyodeoxycholic acid; TMAO: trimethylamine N-oxide; TNF-α: tumor necrosis factor-α; TUDCA: tauroursodeoxycholic acid; UCA: ursocholic acid.

## Data Availability

Not applicable.
